# Travel to malaria-endemic areas: using digital geo-location to assess potential exposure risks and health behaviours

**DOI:** 10.1093/jtm/taae141

**Published:** 2024-10-25

**Authors:** Andrea Farnham, Christoph Hatz, Jan Fehr, Vasiliki Baroutsou, Milo A Puhan, Olivia Veit, Silja Bühler

**Affiliations:** Department of Public and Global Health, Epidemiology, Biostatistics and Prevention Institute, University of Zurich, Hirschengraben 84, 8001 Zurich, Switzerland; Department of Public and Global Health, Epidemiology, Biostatistics and Prevention Institute, University of Zurich, Hirschengraben 84, 8001 Zurich, Switzerland; Center for Tropical and Travel Medicine, Swiss Tropical and Public Health Institute, Allschwil, Switzerland; University of Basel, Basel, Switzerland; Department of Public and Global Health, Epidemiology, Biostatistics and Prevention Institute, University of Zurich, Hirschengraben 84, 8001 Zurich, Switzerland; Department of Public and Global Health, Epidemiology, Biostatistics and Prevention Institute, University of Zurich, Hirschengraben 84, 8001 Zurich, Switzerland; Department of Public and Global Health, Epidemiology, Biostatistics and Prevention Institute, University of Zurich, Hirschengraben 84, 8001 Zurich, Switzerland; Center for Tropical and Travel Medicine, Swiss Tropical and Public Health Institute, Allschwil, Switzerland; University of Basel, Basel, Switzerland; Division of Tropical and Humanitarian Medicine, Geneva University Hospitals, Geneva, Switzerland; Department of Public and Global Health, Epidemiology, Biostatistics and Prevention Institute, University of Zurich, Hirschengraben 84, 8001 Zurich, Switzerland; Division of Hygiene and Infectious Diseases, Institute of Hygiene and Environment, Hamburg, Germany

**Keywords:** mHealth, malaria, malaria risk, travel medicine, health behaviour, TOURIST2

## Abstract

**Background:**

Travellers frequently visit popular destinations like Brazil, India, Peru, Thailand and Tanzania, each presenting varying malaria risks. The extent to which travellers enter high-risk malaria-endemic areas in destinations with heterogeneous malaria risk remains unclear. We used geo-location via a smartphone application to (i) describe where travellers go within countries with heterogeneous malaria risk (Brazil, India, Peru, Thailand) and (ii) compare mosquito bite prevention behaviours between these destinations and Tanzania, considered entirely high risk for malaria.

**Methods:**

This analysis is a sub-study of the Tracking of Urgent Risks in Swiss Travellers (TOURIST2) cohort, which prospectively recruited 1000 travellers (≥18 years, travelling ≤4 weeks) from Swiss travel clinics (Zurich and Basel) between September 2017 and April 2019. We included 734 travellers to Brazil, India, Peru, Thailand and Tanzania who provided geo-location data. Daily health and geo-location data were collected using a smartphone application. Malaria risk was categorized using 2022 malaria maps from the Swiss Expert Committee for Travel Medicine.

**Results:**

Of the 734 travellers, 525 travelled to Brazil, India, Peru and Thailand and 225 to Tanzania. In Brazil, India, Peru and Thailand, only 2% (*n* = 13) visited high-risk malaria areas. In Peru, 4% (*n* = 4) visited a high-risk area; in Brazil, 3% (*n* = 6); in Thailand, 2% when crossing the border into Myanmar (*n* = 3); and in India, 0%. Travellers to high-risk areas were more often male (62%), slightly older (median age 42.0) and planned longer trips (median 23.0 days) than other travellers. No participants were diagnosed with malaria. Travellers to Brazil, India, Peru and Thailand used mosquito bite prevention measures less frequently than travellers to Tanzania. Those in Tanzania had higher, but still suboptimal, use of insect spray (65% of travel days).

**Conclusions:**

Travellers to Brazil, India, Peru and Thailand rarely visited high-risk malaria areas, and their adherence to mosquito bite prevention measures was generally low. In Tanzania, adherence was higher but still suboptimal.

## Background

Malaria continues to be a global health priority. Despite widespread progress in malaria control internationally, increasing numbers of travellers from non-malaria-endemic countries visit malaria-endemic regions.^1–4^ Travel-related malaria cases continue to occur and often result in poor health outcomes and high mortality, as well as high costs to returning travellers.^1^^,^^2^^,^^5^^,^^6^ Malaria remains one of the most important and dangerous infections faced by travellers,^5^^,^^7–9^ although the majority of travellers to endemic regions do not contract it.^10^

The risk to travellers of exposure to malaria during travel, especially to destinations with heterogeneous malaria risk, remains a controversial issue.^11^ Experts have suggested that the exposure risk depends on where travellers go within the destination country, the degree of endemic malaria transmission in that region, the duration and style of travel and the use of malaria preventive measures.^12^^,^^13^ There is evidence that certain groups seem to be at higher risk of exposure, such as those travelling to Sub-Saharan Africa, those visiting friends and relatives and children.^2^^,^^9–11^^,^^14^ Malaria preventive measures include mosquito bite prevention behaviours (e.g. insect spray, insecticide, bed nets, long insecticide-treated clothing) and chemoprophylaxis, depending on the degree of risk of malaria to the traveller.^14^^,^^15^ Travel medicine practitioners take all these individual factors into account when recommending malaria prevention measures to travellers to malaria-endemic regions.^11^^,^^13^

Little is still known about the degree to which travellers visit areas considered to be high risk (i.e. with high local *Plasmodium falciparum* incidence) in countries where the malaria risk level varies geographically, such as in popular tourist destinations like Brazil, India, Peru and Thailand. This leads to considerable uncertainty and debate around recommendations for chemoprophylaxis for travel medicine practitioners advising visitors to these countries, with different advisory bodies globally providing different guidance.^13^ It is also unknown whether travellers adhere to the behavioural advice on mosquito bite prevention measures that are given at the travel medicine consultation,^7^ especially in countries where the risk of malaria is region-dependent and therefore awareness of the risk of malaria may be lower.

Digital tools such as travel smartphone applications offer new opportunities to geo-locate traveller itineraries in real time, linking environmental exposures, health behaviours and health outcomes.^16^ Some travel medicine research groups have developed smartphone applications that specifically track traveller location, health, behaviours and environment during their trips, including the TOURIST studies.^17–22^ Whether traveller compliance with mosquito bite prevention measures varies depending on where they travel in countries with regional variations in malaria risk is still unknown. The degree to which mosquito prevention measures are adhered to by travellers in heterogeneous malaria-risk countries in comparison with holoendemic countries (e.g. Tanzania) is also unknown.

The aim of this study was to use digital data from a travel smartphone application to (i) describe where travellers go in countries with heterogeneous malaria risk (i.e. Brazil, India, Peru and Thailand) and (ii) compare mosquito bite prevention behaviours between travellers to countries with heterogeneous malaria risk and travellers to Tanzania, a country with high malaria risk throughout the country.

## Methods

### Study population and design

This study is a sub-analysis of the longitudinal TOURIST2 cohort study. The TOURIST2 study methodology has been described in detail elsewhere^17^^,^^22^; in short, we recruited 1000 travellers from travel clinics in Basel and Zurich, Switzerland, who were travelling to six popular destinations (Brazil, India, Peru, Thailand, Tanzania). In the present sub-study, we included travellers to five of these countries, including four destinations (Brazil, India, Peru, Thailand) with heterogeneous malaria risk and one (Tanzania) with high malaria risk throughout. Tanzania was included as a comparison point for mosquito bite prevention behaviours in holoendemic versus heterogeneous risk countries. China was excluded, as it was certified malaria-free in 2021 after four consecutive years of zero indigenous cases. Travellers were eligible to participate if they were aged ≥18 years, intended to travel for ≤4 weeks, were able to use a smartphone during their trips and were planning to visit one of the study countries between September 2017 and April 2019. In the present sub-analysis, we excluded travellers without any geo-location data.

As part of the TOURIST2 study, travellers were asked to (i) complete a pre-travel questionnaire with basic demographic, travel and medical information; (ii) complete a daily electronic questionnaire via the study smartphone application on their health events and risk behaviours during the past 24 hours for 10 days at home prior to their trip, (iii) each day during the trip, and (iv) for 14 days at home after their trip; and (v) complete an electronic post-travel questionnaire on their health after return. Participants were also informed that the study smartphone application would automatically collect data on their itinerary and location during their travels. Travellers were considered participants in the study when they submitted at least one survey during travel and did not withdraw consent. Participants received a standard pre-travel consultation prior to their trips. To incentivize participation, participants received a free travel medicine consultation and a subscriber identity (SIM) card that would provide free internet on their smartphone during travel to the study country. All participants provided informed consent electronically.

### Data collection with the TRAVEL smartphone application

The TRAVEL (Tracking Risks Abroad on Vacation with Electronic Localisation) smartphone application was developed during the TOURIST1 study in partnership with the Wearable Computing Lab of the Swiss Federal Institute of Technology (ETH Zurich, Switzerland).^17–22^ The study application collected passive data on traveller geo-location automatically every 15 minutes via a global positioning system (GPS) fix and collected active data via the daily electronic questionnaire. Travellers were prompted to complete the questionnaire daily in the evening via a push notification on their phone. In this sub-analysis, we used only the GPS data, data on bite prevention behaviours and itchiness from mosquitoes from the daily questionnaire and data from the pre-travel questionnaire.

Every 15 minutes, the application automatically initiated a search for location; if successful, this search generated one geo-located point. If internet access was available, either through Wi-Fi or the study SIM card, the participant’s location was identified through triangulation of nearby Wi-Fi access points or cell phone towers via web-based lookup. If no internet access was available, localization was obtained through a GPS fix via satellite connection. If GPS connection was not available (usually indoors or in densely populated areas), Wi-Fi or cell tower localization was used instead, with a corresponding loss in geographical precision of 50 m to a kilometre. To allow for filtering inaccurate location data when appropriate, an error estimation of the positioning has been recorded. Location and new questionnaire data were transmitted to study servers every hour. If a connection to the study servers was unavailable, the transmission was retried hourly until it was successful.

Management of the baseline demographic and health data was done using the Research Electronic Data Capture (REDCap) database software. Location and questionnaire data from the TRAVEL application were exported from the study servers via the web front-end as a comma-delimited (csv) file and merged with the baseline data using the anonymous study pseudonym.

### Mapping malaria risk

To identify visits to malaria risk areas, individual traveller paths were mapped over malaria risk maps from 2022, established by the Swiss Expert Committee for Travel Medicine (www.healthytravel.ch). The maps from 2022 were chosen because prior to this year, geographically detailed risk maps for travellers were not available from the Swiss Expert Committee for Travel Medicine (general risk maps were used for entire countries). The categorization of malaria risk areas is described in the 2022 guidelines for the Swiss Expert Committee for Travel Medicine.^23^ In these maps, the risk category definitions were based on the annual reported number of malaria cases (all species) in endemic areas per 1000 local population. Risk categories were defined as ‘high’ if >10 malaria cases per 1000 population were recorded, ‘low’ if 1–10 cases per 1000 population, ‘minimal’ if <1 case per 1000 local population and ‘none’ if zero cases per 1000 population per year were recorded. Data were sourced from the World Malaria Reports of 2019, 2020 and 2021. The highest recorded incidence of the three reports was used. In the present study, countries with more than one risk category are defined as countries with ‘heterogeneous malaria risk’ (i.e. Brazil, India, Peru, Thailand), while Tanzania including the Zanzibar archipelago is defined as a ‘uniformly high malaria risk’ country.

First, the 2022 maps were georeferenced within Quantum Geographic Information System (QGIS) software using Natural Earth maps. Each individual traveller GPS data point was categorized as ‘high’, ‘low’, ‘minimal’ or ‘no risk’ based on the 2022 malaria risk map for the corresponding country. For each risk category and destination country, we determined the number and proportion of travellers who visited areas with each level of malaria risk. In countries with heterogeneous malaria risk (Brazil, India, Peru, Thailand), we further classified travellers into two groups: those who visited high-risk malaria areas and those who only visited lower-risk areas, to enable a comparison of characteristics. Since not all travellers provided geo-located data for the entirety of their trip, it was not feasible to calculate the exact duration each traveller spent in high-risk malaria areas. Instead, we calculated the proportion of GPS data points collected within high-risk malaria zones for each destination country, which serves as a proxy for the time spent in these areas across all travellers.

### Mosquito bite prevention behaviours

Travellers were asked each evening during their trip which of the following mosquito bite prevention behaviours that they had used that day: (i) insect spray used on the skin, (ii) insecticide used in the room or on clothing, (iii) clothing that covered their arms and legs, (iv) socks or closed toe shoes and (v) mosquito protection overnight (e.g. mosquito net, screen or air conditioning). While travellers were prescribed malaria chemoprophylaxis during their travel consultation if they planned on travelling to a malaria-risk area, compliance was not tracked by the application.

### Statistical analysis

Descriptive statistics were calculated to compare the demographic characteristics and prevalence of different health behaviours in the different risk groups, including medians and interquartile ranges (IQRs) for continuous data and percentages for categorical data.

To calculate the incidence of mosquito bite prevention behaviours by country, the number of mosquito bite prevention behaviours was summed up by day and traveller and then divided by the total number of geo-tagged survey days for that country and multiplied by 1000 to obtain the incidence per 1000 travel days. All analyses were conducted with R version 4.2.3.^24^

The study was approved by the Ethics Commission of the Canton of Zurich (KEK-ZH-Nr. 2014–0470, BASEC-Nr. PB_2017–00412). The TOURIST2 study is registered with clinicaltrials.gov under the identifier NCT03262337.

## Results

### Study population

Of the 1000 eligible travellers enrolled in the entire TOURIST2 study, 793 (79%) completed the study. Of those, 26 were travelling only to China, which was not considered a malaria-risk destination, and were therefore excluded from the analysis. Of the remaining 767 participants, 734 (96%) were able to be geo-located during travel and therefore included in this study (Tables 1 and 2). Characteristics of our study population are shown in Table 2. In brief, study participants were a median of 34.0 years of age at enrolment (IQR: 27.0–49.0) and 55% female. Travellers planned trips of median 16.0 days (IQR: 14.0–23.0). Travellers filled out a median of 11 surveys during their trips, for a median participation rate of 79%. For characteristics of the entire TOURIST2 study population, please see previous publications.^17^^,^^22^

**Table 1:** Geographical data collected on participants in countries with heterogeneous malaria risk. Geo-located points were captured every 15 minutes; more geo-located points correspond to a higher proportion of time spent in that area

	Brazil	India	Thailand	Peru
Number of travellers	183	145	135	95
Number of travellers with GPS information (%)^a^	178 (97%)	142 (98%)	129 (96%)	89 (94%)
Number of geo-located points	972 862	625 032	685 160	263 921
Number of geo-located points within high-risk malaria areas (%)	178 (0.02%)	0 (0%)	30^b^ (0.004%)	523 (2%)
Number of travellers visiting an area with any malaria risk (%)^a^	34 (19%)	142 (100%)	129 (100%)	35 (39%)
Number of travellers in malaria risk areas^a^				
High risk	6 (3%)	0 (0%)	3^b^ (2%)	4 (4%)
Low risk	24 (13%)	5 (4%)	3 (2%)	5 (6%)
Minimal risk	20 (11%)	141 (99%)	129 (100%)	29 (33%)

^a^Participants could visit more than one type of risk area; therefore, columns sum up to more than total number of participants.

^b^Thailand travellers only encountered high-risk areas when travelling along and over the border with Myanmar.

**Table 2:** Characteristics of the entire study population and those that visited malaria-endemic areas [*n* (%) or median (IQR)]

			Countries with heterogeneous malaria risk (Brazil, India, Peru, Thailand)				Tanzania
	Overall study	Overall study with geo-located data	No malaria risk	Malaria visits in heterogeneous countries			Uniformly high risk for malaria
				High risk	Low risk	Minimal risk	High risk
Participants^a^	767	734 (96%)	267	13	37	314	215
Female sex	421 (55%)	402 (55%)	139 (52%)	5 (38%)	20 (54%)	180 (57%)	117 (54%)
Age	34.0 (27.0, 50.0)	34.0 (27.0, 49.0)	33.0 (28.0, 47.5)	42.0 (35.0. 45.0)	33.0 (28.0, 43.0)	32.5 (27.0, 48.0)	37.0 (28.0, 51.0)
Planned trip days	16.0 (14.0, 23.0)	16.0 (14.0, 23.0)	18.0 (14.0, 23.0)	23.0 (21.0, 27.0)	22.0 (15.0, 25.0)	19.0 (15.0, 26.8)	15.0 (12.0, 19.0)
Reason for travel							
Holiday	601 (78%)	573 (78%)	200 (75%)	10 (77%)	31 (84%)	250 (80%)	177 (82%)
Business	55 (7%)	54 (7%)	27 (10%)	3 (23%)	3 (8%)	19 (6%)	13 (6%)
Volunteering	28 (4%)	28 (4%)	4 (1%)	0	0	11 (4%)	15 (7%)
Visiting friends and relatives	62 (8%)	59 (8%)	34 (13%)	0	3 (8%)	18 (6%)	7 (3%)
Missing	21 (3%)	20 (3%)	2 (0.7%)	0	0	16 (5%)	3 (1%)
Total survey days^b^	15 455	14 850	5179	413	943	7094	4060
Total survey days in-country^c^	9276	8910	3303	234	564	4240	2215
Used insect spray for skin (survey days)	4924 (53%)	4703 (53%)	1209 (37%)	85 (36%)	258 (46%)	2314 (55%)	1450 (65%)
Used insecticide for clothing or in the room (survey days)	2003 (22%)	1900 (21%)	382 (12%)	29 (12%)	87 (15%)	865 (20%)	734 (33%)
Used clothing that covered arms and legs (survey days)	2802 (30%)	2665 (30%)	625 (19%)	87 (37%)	147 (26%)	1199 (28%)	1017 (46%)
Used socks or closed-toe shoes (survey days)	2883 (31%)	2717 (30%)	783 (24%)	100 (43%)	205 (36%)	1131 (27%)	1032 (47%)
Used mosquito bite prevention during the night (survey days)	3636 (39%)	3464 (39%)	519 (16%)	40 (17%)	126 (22%)	1544 (36%)	1509 (68%)
Reported noticing mosquito bites (survey days)	1446 (16%)	1415 (16%)	554 (17%)	27 (12%)	118 (21%)	682 (16%)	298 (13%)

^a^Participants could visit more than one type of risk area; therefore, columns sum up to more than total number of participants

^b^Includes entire trip (e.g. participants who visited a high-risk setting could then take malaria post-exposure prophylaxis in a minimal-risk setting, and this would be counted for both). Data from pre-travel were not included.

^c^Includes only travel days in the country and not survey days after home.

### Where did travellers go?

**Figure 1:**
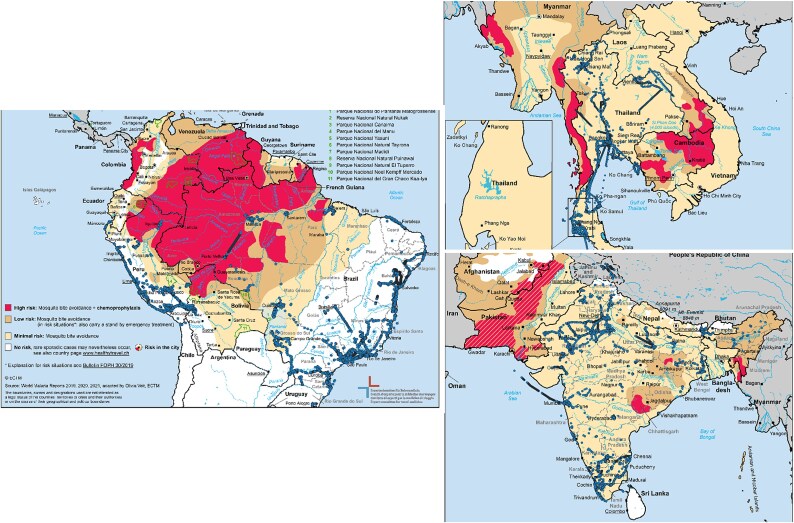
Traveller geo-locations in countries with heterogeneous malaria risk shown as dots; the maps represent the recommendations issued in 2022. The red areas are considered high risk of malaria, the brown areas low risk, the yellow regions minimal risk and the white areas no risk.

In total, 546 travellers visited Brazil, India, Peru or Thailand, the countries with heterogeneous malaria risk. Of these, 94 travellers (17%) visited more than one of these countries. Of the 546 total travellers, 525 (96%) were able to be geo-located during their trips (Table 1). The exact locations of the travellers visiting Brazil, India, Peru and Thailand are shown in Fig. 1.

**Figure 2:**
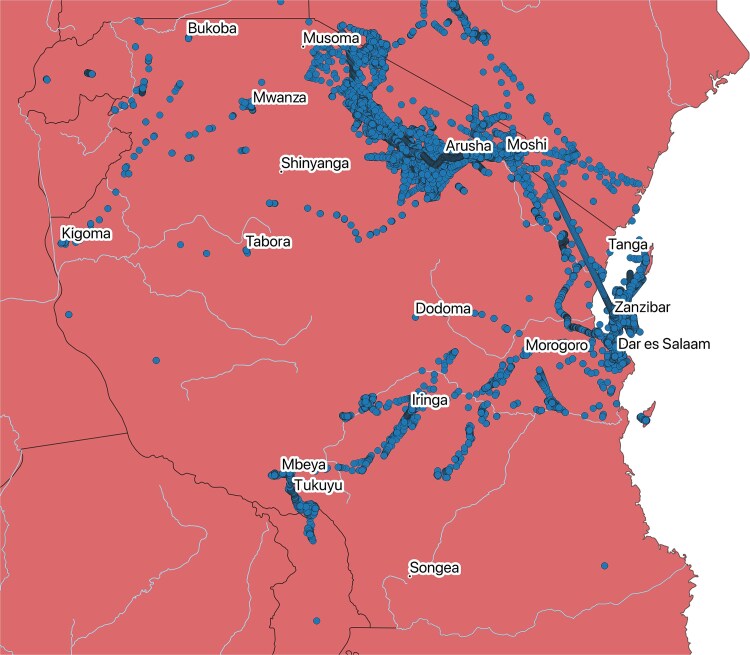
Traveller geo-locations in Tanzania shown as dots. The entirety of Tanzania is considered to have a high risk of malaria.

Two hundred twenty-five travellers in total visited Tanzania, of which 215 (96%) were able to be geo-located during their trips. The exact locations of travellers to Tanzania are shown in Fig. 2.

### Visits to high-risk malaria areas in Brazil, India, Peru and Thailand

Of the 525 travellers that were able to be geo-located within heterogeneous malaria-risk countries, 13 (2%) were detected visiting high-risk malaria areas. This included four travellers (4%) in Peru, six (3%) in Brazil and none in India (Table 1). Three travellers to Thailand (2% of Thailand travellers) encountered high-risk malaria areas when travelling over the border with Myanmar. In Peru, 54 participants (61%) and in Brazil, 144 participants (81%) were not detected visiting any areas with malaria risk. Among travellers to Thailand and India, 100% visited an area with some malaria risk, as there are no areas in these countries judged to be of no malaria risk at all (Table 1, Fig. 1). The number of geo-located points located in high-risk malaria areas ranged from 523 location points (2% of total location data) in Peru, 178 location points (0.02%) in Brazil, 30 location points (0.004%) for the travellers to Thailand that crossed the border to Myanmar, to none in India (Table 1).

### Personal characteristics and malaria-protective behaviours

The characteristics of those who visited high-risk malaria areas in Brazil, India, Peru and Thailand differed from those who only visited no-, minimal- or low-risk malaria areas in these countries, although the numbers visiting high-risk areas in these countries were small (*n* = 13/525, 2% of travellers to these countries). Compared to those travelling to lower-risk areas, high-risk travellers tended to be more often male (62% versus 45% overall), older (median age 42.0 versus 34.0 overall), planned longer trips (median 23.0 days versus 16.0 overall) and were travelling more often for business (23% (*n* = 3/13) versus 7%), although most were still travelling on holiday (77%) (Table 2). They reported a mix of travel styles, from luxury hotels to backpacking, but six (46%) reported planning on staying at least one night in hostel/backpacking-style accommodations. Two travellers (15%) reported boat cruises on the Amazon River. They were less likely than lower-risk travellers to regularly use insect spray or insecticide, but they were more likely than lower-risk travellers to use protective clothing that covered their arms and legs and to wear closed-toed shoes and socks (see Table 2). They also used mosquito bite prevention measures during the night on only 17% of survey days. However, these high-risk travellers reported noticing mosquito bites on only 12% of survey days, the lowest rate of all the risk groups (Table 2).

The characteristics of the 215 travellers who visited Tanzania did not differ substantially from the 525 travellers who visited countries that are of heterogeneous malaria risk (Brazil, India, Peru and Thailand, see Table 2). They were slightly older (median age 37.0 vs. 33.0 overall in the four heterogeneous countries) and planned slightly shorter trips (median 15.0 days vs. 18.0). Tanzanian travellers showed a much higher proportion of travel days where they used prevention measures against mosquito bites than travellers to the four countries with heterogeneous risk (Table 2). Tanzanian travellers used insect spray on their skin for 65% of travel days, insecticide-treated clothing or insecticide-spraying in rooms for 33% of travel days and mosquito bite prevention during the night for 68% of travel days. Tanzanian travellers reported noticing fewer mosquito bites (13% of travel days) than travellers to lower-risk areas in heterogeneous risk countries (Table 2).

While travellers who went to high-risk malaria areas in Tanzania showed increased use of prevention measures against mosquito bites, the incidence of mosquito bite prevention behaviours was generally low among all travellers. The most common bite prevention behaviour overall was using insect spray for skin, which was used in 53% of all travel survey days. The usage of insect spray for skin was highest among travellers to Tanzania (65% of travel days) and lowest among travellers to high-risk areas in the four heterogeneous risk countries (36% of travel days). Participants used insecticide on clothing or in the room on 21% of travel days. Travellers reported using clothing that covered their arms and legs on 30% of travel days and using socks and closed-toe shoes on 30% of travel days. Mosquito bite prevention measures during the night were used on 39% of travel days. Participants reported noticing mosquito bites on 16% of travel days. None of the travellers reported a diagnosis of malaria during or after the study.

As shown previously,^17^ when broken down by country, Tanzania had the highest incidence of mosquito bite prevention behaviours by far, with an average incidence of 2603.5 protective behaviours per 1000 survey days (meaning an average of 2.6 of the above protective behaviours by day). This was followed by Thailand (1830 per 1000 survey days), India (1758 per 1000 survey days), Brazil (1127.3 per 1000 survey days) and finally Peru (1059.1 per 1000 survey days).

## Discussion

Our study provides new evidence on the patterns in travel destinations and health behaviour of travellers to malaria-endemic countries. Importantly, we show that travel to areas considered to be high risk in countries with heterogeneous malaria risk such as Brazil, India, Peru and Thailand is rare in this cohort, ranging from 4% of travellers to Peru, 3% to Brazil, to no travellers to India. In Thailand, 2% (*n* = 3) of travellers went over the border to Myanmar and encountered high-risk malaria areas there. In Peru and Brazil, most travellers did not visit any region with malaria risk (61% and 81%, respectively). Compared to the general study population, the small number of travellers (*n* = 13) that did visit high-risk malaria in these countries tended to be more often male (62%), slightly older (median age 42.0), planned longer trips (median 23.0 days), and were travelling more often for business (although the majority were travelling on holiday). Travellers to Brazil, India, Peru and Thailand used mosquito bite prevention measures much less frequently than travellers to Tanzania, but, even in Tanzania, travellers used insect spray on only 65% of travel days. Worryingly, participants reported noticing mosquito bites on 16% of travel days overall during the study, and this proportion was only slightly lower in high-risk malaria areas (13% in Tanzania and 12% in Brazil/Peru/Thailand) than in no-risk malaria areas (17%).

These results suggest that in countries with heterogeneous malaria risk such as Brazil, India, Peru and Thailand, most travellers may only visit areas considered to be at no to minimal risk. Very few travellers travelled to high-risk (*n* = 13/525, 2%) or low-risk (*n* = 37/525, 7%) areas in Brazil, India, Peru and Thailand. More travelled to minimal-risk areas (*n* = 314/525, 60%). This may reflect the fact that high-risk areas tend to be more remote and therefore more difficult to access for the average traveller.

It is important to note that the endemic situation in each country and therefore chemoprophylaxis recommendations can and do change over time, and, therefore, the ‘risk maps’ represent a snapshot of malaria risk at that time point. The most substantial change in recommendations over the study period was for travellers staying only in Zanzibar, an archipelago off the coast of Tanzania. Before 2019, the risk for travellers who only visited Zanzibar or Dar es Salaam was considered to be low by the Swiss Expert Committee for Travel Medicine (ECTM). In 2019, the ECTM elevated the malaria risk to high for the entire country, including Zanzibar and Dar es Salaam, due to an increase in local cases. The maps in this analysis were generated using risk maps from 2022,^23^ which were the geographically detailed risk maps closest in time to the study period. For consistency, we also used the 2022 recommendations for Tanzania. If we perform the analysis instead of using the 2023 ECTM risk maps that use a different methodology, there are fewer areas considered to be ‘high risk’ in the heterogeneous malaria-risk countries. Using the 2023 maps, there are no longer any high-risk travellers to Brazil, leaving only the four high-risk travellers in Peru. Therefore, even the low numbers in this study can be considered an upper estimate of the number of travellers in this cohort who encountered high-risk malaria settings in the countries with heterogeneous malaria risk.

Of more concern is the relatively low rate of mosquito bite prevention measures reported by all travellers. Mosquito bite prevention behaviours varied widely by country, indicating that risk perception is very different in travellers to different destinations. As most imported malaria cases come from Sub-Saharan Africa,^1^^,^^8^ travellers may be more conscious of using mosquito bite prevention, or travel medicine practitioners may emphasize the importance of mosquito bite prevention more to these travellers. It is therefore unsurprising that travellers to Tanzania showed the highest incidence of mosquito bite prevention behaviours. It is of note that travellers to high-risk malaria areas in all countries reported noticing the fewest mosquito bites. More surprisingly, high-risk travellers to heterogeneous countries largely had the lowest rate of insect spray use, instead seeming to rely on the widespread use of long pants and shirts and closed-toed shoes. Without qualitative follow-up, it is difficult to ascertain why travellers to these areas used less insect spray than those to Tanzania or lower-risk areas. Previous literature has suggested that certain risk groups, like those travelling to visit friends and relatives, may be less likely to use preventive measures against malaria, although this was mostly in Sub-Saharan Africa.^2^ More follow-up research should be done to determine how and why different travellers choose different methods of mosquito bite prevention and how to promote effective mosquito bite prevention behaviour even outside of malaria-endemic areas.

It is crucial to note that there are important mosquito-borne diseases in these heterogeneous-malaria risk destinations other than malaria (e.g. dengue, chikungunya, Zika, Japanese encephalitis, yellow fever) and that advice during the pre-travel consultation should not focus solely on malaria prevention. Brazil and Peru travellers were at risk of yellow fever, dengue, chikungunya and Zika, depending on where they travelled. In Thailand and India, there is a risk of dengue, chikungunya, Zika and Japanese encephalitis (although the risk is generally considered very low for most travellers). Previous research has shown that the risk of different mosquito-borne diseases varies by destination, with disproportionate numbers of malaria cases in travellers returning from Africa, dengue in travellers returning from Southeast Asia and Latin America and the Caribbean and chikungunya in travellers returning from Southeast Asia.^25^^,^^26^ Some research has even suggested that arbovirus diagnoses are becoming more common among travellers than malaria diagnoses.^26^ The mosquito species that transmit these diseases behave differently, which influences the timing and type of protective measures recommended.^27^ For example, while malaria prevention often focuses on evening and nighttime protection due to the nocturnal feeding habit of Anopheles mosquitoes, diseases like dengue require daytime protection due to the differing behaviours of the Aedes mosquitoes.^27^ The local trends in the epidemiology of these diseases also play an important role in advice to travellers, as importation trends in returned travellers often mirror local outbreaks in destination countries.^28^ The low adherence to preventative measures, even in non-malaria areas, suggests a significant gap in travellers’ understanding of the risks posed by these diseases. Future research should explore strategies to improve awareness and prevention of mosquito-borne diseases beyond malaria.

This study has limitations. The findings may not be generalizable to all travellers, as most participants (79%) were tourists on short-term trips (median 16.0 days). Other traveller groups, such as those visiting family and friends, volunteers, non-Swiss or long-term travellers or those that do not seek out a pre-travel consultation, may be more likely to visit high-risk malaria areas. Furthermore, the study only tracked travellers to four countries with heterogeneous risks, so the findings may not apply to other destinations with similar risk profiles. Another limitation is that we used detailed risk maps for malaria for the study countries from 2022, while data collection occurred between 2017 and 2019. The completeness of the geo-location data needs to be interpreted with caution. Participants may have turned off geo-location on their phones to save battery, particularly in remote areas, or geo-location may not have functioned as well in these areas. Geo-location data were provided every 15 minutes but surveys only once a day (and less frequently in some cases), meaning that mosquito bite prevention behaviours and health outcomes cannot be precisely geo-located to each high-risk area (except in the case of Tanzania). Surveys were sometimes not uploaded at the point where they were entered, depending on internet connectivity; therefore, we can only report general trends in behaviours. Nevertheless, this study provides greater granularity on travel patterns and health behaviours during travel in malaria-endemic areas than in previous studies, to our knowledge. The data were collected pre-pandemic, and consciousness of certain infectious diseases may have changed in the post-pandemic era, although it is unlikely that tourist itineraries have changed substantially. Finally, the proportion of travellers to each country that was prescribed and took malaria chemoprophylaxis would be a valuable addition to the discussion around malaria prevention behaviour during travel, but unfortunately, these data were not collected by the app. Previous studies that explored compliance with malaria prophylaxis during travel found that non-compliance was frequent (20% of travellers).^29^

## Conclusions

Travellers rarely visited high-risk malaria-endemic areas in countries with heterogeneous risk in this cohort. Compliance with mosquito bite prevention measures was also low. Travellers to Tanzania, a country with high malaria risk throughout the country, had higher but not optimal compliance with mosquito bite prevention measures.

## Data Availability

The data supporting this study's findings are not publicly available due to their detailed and sensitive nature. However, data can be made available by the corresponding author upon reasonable request, provided that any applicable privacy and ethical considerations are met.
